# Influence of age, socioeconomic status, and location on the infant gut resistome across populations

**DOI:** 10.1080/19490976.2023.2297837

**Published:** 2024-01-13

**Authors:** Dhrati V. Patangia, Ghjuvan Grimaud, Shaopu Wang, R. Paul Ross, Catherine Stanton

**Affiliations:** a School of Microbiology, University College Cork, Cork, Ireland; b Teagasc Food Research Centre, Moorepark, Fermoy, Co. Cork, Ireland; c APC Microbiome Ireland, Cork, Ireland; d Key Laboratory of Birth Defects and Related Diseases of Women and Children (Sichuan University), Ministry of Education, Department of Pediatrics, West China Second University Hospital, Sichuan University, Chengdu, China

**Keywords:** Infant gut resistance, meta-analysis, shotgun sequencing, resistome

## Abstract

Antibiotic resistance is a growing global concern, with many ecological niches showing a high abundance of antibiotic resistance genes (ARGs), including the human gut. With increasing indications of ARGs in infants, this study aims to investigate the gut resistome profile during early life at a wider geographic level. To achieve this objective, we utilized stool samples data from 26 studies involving subjects aged up to 3 years from different geographical locations. The 32,277 Metagenome Assembled Genomes (MAGs) previously generated from shotgun sequencing reads from these studies were used for resistome analysis using RGI with the CARD database. This analysis showed that the distribution of ARGs across the countries in our study differed in alpha diversity and compositionally. In particular, the abundance of ARGs was found to vary by socioeconomic status and healthcare access and quality (HAQ) index. Surprisingly, countries having lower socioeconomic status and HAQ indices showed lower ARG abundance, which was contradictory to previous reports. Gram-negative genera, including *Escherichia, Enterobacter, Citrobacter*, and *Klebsiella* harbored a particularly rich set of ARGs, which included antibiotics that belong to the Reserve, Access or Watch category, such as glycopeptides, fluoroquinolones, sulfonamides, macrolides, and tetracyclines. We showed that ARG abundance exponentially decreased with time during the first 3 years of life. Many highly ARG-abundant species including *Escherichia, Klebsiella, Citrobacter* species that we observed are well-known pathobionts found in the infant gut in early life. High abundance of these species and a diverse range of ARGs in their genomes point toward the infant gut, acting as an ARG reservoir. This is a concern and further studies are needed to examine the causal effect and its consequences on long-term health.

## Introduction

Early life gut microbial colonization has been studied extensively, thanks to advancements in meta-genomic sequencing technologies.^1^ Increasing advances and decreasing costs have resulted in several studies identifying and establishing the role and importance of early-life gut microbiota colonization in health, innate and adaptive immunity development, and disease development in later life.^2–5^ The infant gut microbiota is highly dynamic and stabilized by the age of 3 years. In the past decade, several studies have delineated the effects of factors such as mode of delivery, feeding habits, gestational age, environmental factors, and antibiotic consumption on early life microbial colonization.^4–8^ Antibiotics are among the most frequently prescribed drugs for infants, with studies reporting frequent and pro-longed antibiotic use and misuse in neonates and children prophylactically, and for precise reasons such as specific treatments.^9,10^ Several adverse effects, including altered initial gut microbial colonization, which increases the likelihood of allergic and metabolic disorders later in life,^11^ accompany the life-saving benefits of antibiotics. The worst adverse outcome of antibiotic use is the development of antibiotic resistance among the gut microbiota; indeed, antibiotic resistance is considered a global threat and is responsible for the death of hundreds of thousands of people each year^12–14^ reported to reach an estimated 10 million deaths by 2050.^15^

The number of studies reporting the presence and source of antibiotic resistance genes (ARGs) in infants has increased drastically owing to the dire situation created by the increasing spread, abundance, ubiquitous nature of ARGs and the high abundance of ARGs reported in Low – to Middle – income countries.^16,17^ Recent studies have shed light on ARG abundance and the extent to which several factors affect it.^16,18–20^ Studies have demonstrated a high abundance of ARGs in infants, even in the absence of antibiotic exposure, making the infant gut a reservoir of ARGs.^6,21–23^ This is a serious concern, as ARGs can be transmitted within bacterial communities by horizontal gene transfer, the possibility of which is high in a densely populated environment such as the gut. Furthermore, ARGs can be transferred between bacteria from different species using mobile genetic elements and, in some cases, from antibiotic-producing bacteria using conjugative methods.^24–27^ The use of antibiotics in infancy disturbs microbial composition, results in high antibiotic resistance, and can positively select taxa with a high abundance of ARGs. Studies have also reported that bacteria belonging to Gammaproteobacteria have the highest abundance of ARGs^16,28,29^, and several taxa within this group are pathobionts or potential pathogens. Infants can also acquire anti-biotic-resistant strains from mothers through vertical transmission.^30^ These resistant bacteria increase the chances of disease, resulting in harder-to-treat infections later in life, increase the cost of medical care, and can result in mortality.^14,15,31^

Reports on the presence and persistence of ARGs in the antibiotic-naïve infant gut suggest the complex nature of this issue, and it has been acknowledged that factors, including antibiotic exposure, age of life, feeding pattern, and delivery mode, affect microbial composition and, therefore, the resistome profile.^16,18–20^ Geographic location is another factor that affects microbiota development; however, it has not been comprehensively associated with the infant gut ARG profile. We thus hypothesized that the infant gut resistome profile could vary as the infant grows because of different environmental factors, risk of infections, and evolving microbial colonization that can modify the resistome repertoire by adding or removing ARGs. To address this issue, we performed a meta-analysis by selecting studies that included metagenomic shotgun sequencing data of infants under 3 years of age across populations. We then examined the effects of geography, delivery mode, age of life, socioeconomic status, healthcare access and quality index (HAQ) on the infants’ resistome profile and provided a comprehensive picture of the ARG burden in the infant gut on an international scale. Such monitoring will provide a representation of the ARG pool in infant gut and help to strategize antibiotic use and its alternatives.

## Results

In our aim to examine the resistome profile of infants in early life, we identified 26 studies to be included in our meta-analysis post duplicate removal, that fit our inclusion criteria. The meta-analysis included studies conducted across populations analyzing data from 6122 metagenome samples, including North America (samples = 2420; subjects = 1311), the United Kingdom (samples = 1519; subjects = 611), New Zealand (samples = 646; subjects = 210), Europe (samples = 1162; subjects = 386), Asia (samples = 70; subjects = 58), and Russia (samples = 305; subjects = 70), spanning four continents. Of the 6122 infant samples included in the meta-analysis, 43.85% corresponded to Caesarean section and 55.97% to vaginally delivered with no information about 0.18%. The 6122 metagenome samples resulted in the formation of 32,227 early life gut metagenome assembled genomes (ELGGs) published in our previous study.^1^ These metagenome assembled genomes (MAGs) were used to investigate the gut resistome profiles in early life in the present study. Other variables considered include the socioeconomic status of the country and the HAQ index. The HAQ index obtained on a scale of 1–100 was transformed into the following categories: very low/below (≤50), low/below (≤80), and above (>80).

### Antibiotic resistance distribution in infants

**Figure 1:**
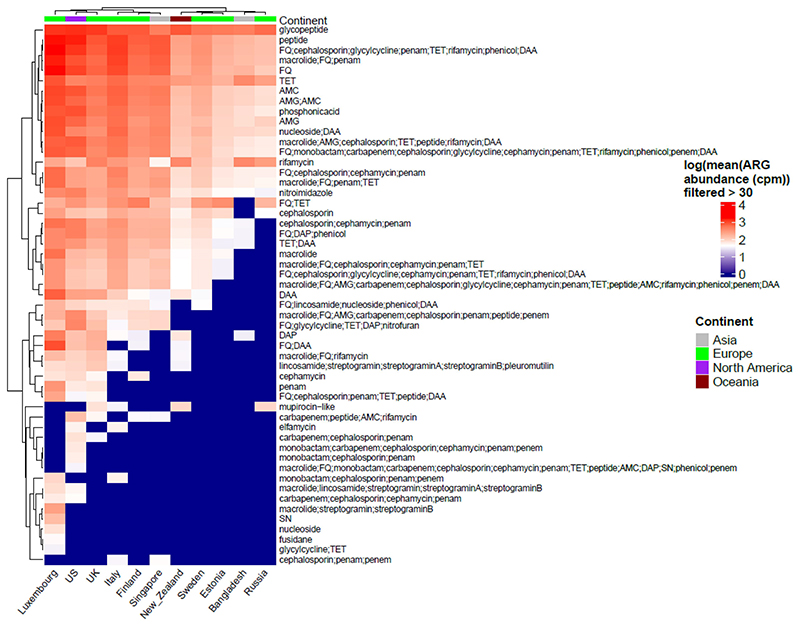
Heatmap showing all the classes of antibiotics per country (we only display here logarithmic mean ARG abundance higher than 30 cpm). Columns in the heatmap represent countries while rows represent antibiotic classes with top row annotation showing continents. Some of the antibiotic classes are abbreviated in the heatmap and are as follows: AMG = “aminoglycoside”, TET = “tetracycline”, RIF = “rifampin”, FQ = “fluoroquinolone”, DAA = “disinfecting agents and antiseptics”, DAP = “diaminopyrimidine”, SN = “sulfonamide” and AMC = “aminocoumarin”.

Of the 6122 samples included, 5886 demonstrated resistance to at least one antibiotic class and were included in the downstream analysis. The presence of 102 different classes of antibiotics, including several multidrug resistance (MDR) classes, was detected using RGI with the CARD database. Antibiotic classes as assigned by RGI with CARD database were used for analysis. MDR assignment was done by manual curation when the ARG conferred resistance to 3 or more different classes of antibiotics. The ARG abundance data per MAG were normalized to copies per million (cpm) using MAG relative abundance data. Resistance to several antibiotics, including glycopeptides, peptides, fluoroquinolones, tetracyclines, macrolides, and rifamycin, was observed in all countries (Figures 1 and S1). Glycopeptides were found to be the most abundant ARG class and were present in all countries, except Singapore. We observed 2 main clusters of antibiotic classes, with antibiotic resistance classes showing low (e.g., fusidane, nucleoside) vs high (e.g., glycopeptide, peptide) abundance in most countries. Similarly, we observed 2 main clusters of countries showing low (e.g., Russia, Bangladesh) vs high (e.g., Luxembourg, USA) abundance of antibiotic resistance classes (Figure 1).

**Figure 2:**
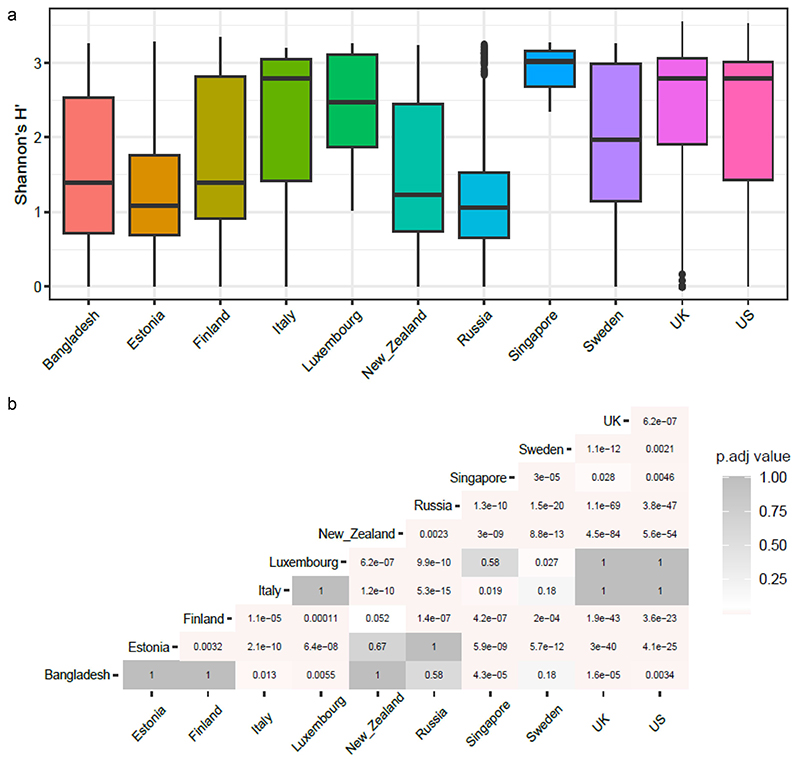
(a) Box plots showing alpha diversity of cpm normalized antibiotic resistance classes relative abundance using Shannon index. (b) Heatmap showing adjusted *p*-values based on alpha diversity pairwise comparisons between countries.

**Figure 3:**
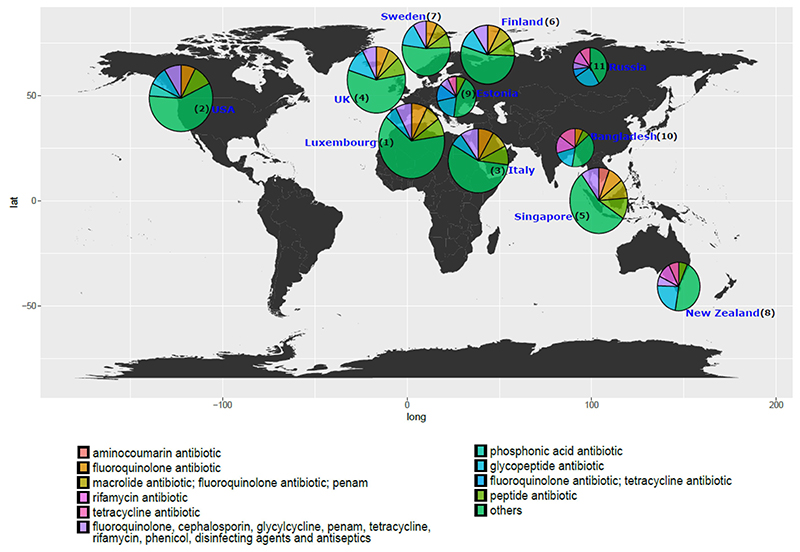
World map showing the top 5 antibiotic resistance classes per country while the remaining classes are represented as “others” in the pie charts for each country. The abundance depicted is normalized to cpm. The radius of each pie corresponds to the (ARG)^1/4^ of the total mean ARG abundance per country. Based on the total ARG abundance per country, we have assigned ranks to the countries (number denoted in parentheses) with rank 1 meaning highest total ARG abundance and rank 11 meaning lowest.

To understand the relationship between antibiotic resistance class diversity and countries included in our study, we looked at alpha-diversity of ARG classes using Shannon index for evenness and richness. We report that countries including Luxembourg and Singapore have high ARG class diversity with low variance (Figure 2a), whereas countries such as the UK, USA, and Italy show high diversity and high variance in the Shannon index. The Shannon index was significantly different between Luxembourg, Singapore, and all other countries and between the UK and USA and the majority of other countries (see Wilcoxon test adjusted *p*-values, Figure 2b). We then looked at the top 5 most abundant antibiotic classes per country and ranked the countries by the mean ARG abundance (Figure 3). All antibiotic classes apart from the top 5 shown were grouped as “others.” The top 5 ARGs observed in all countries provided resistance to fluoroquinolone antibiotics and a MDR class (i.e., which confers resistance to fluoroquinolone, cephalosporin, glycycline, penicillin, tetracycline, rifamycin, phenicol antibiotic, disinfecting agents, and antiseptics). Other classes that are seen in the top 5 are aminocoumarin, macrolide and fluoroquinolone, rifamycin, tetracycline, phosphonic acid antibiotics, fluoroquinolone and tetracycline, and peptide antibiotics. Several of the top 5 most abundant ARGs in infants that we report are known to spread to other bacteria and thus classified as acquired ARGs.^32,33^ Based on our overall ARG analysis (Figure 1), alpha diversity (Figure 3) and top 5 ARG (Figure 3) we report that Luxembourg, the USA, and the UK showed the highest ARG abundance and diversity.

### Most abundant antibiotic resistance classes are carried by gram-negative bacteria

**Figure 4:**
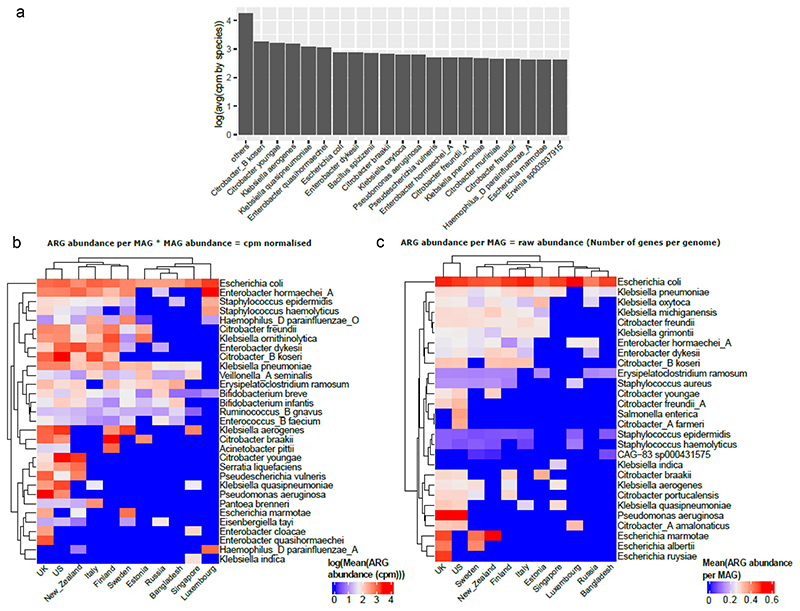
(a) Bar plot representing top 20 species that carry highest ARG abundance globally in infants under 3 years of age using mean of cpm normalized abundance per MAG grouped by species and with the rest of the species represented as “others”. Top 5 species carrying highest ARG abundance per country using (b) cpm normalized data and (c) raw abundance data (i.e., number of ARGs per MAG).

Next, we examined the top 20 species that carried the highest ARG abundance (in cpm values) and found that the majority of these species were gram-negative bacteria (Figure 4a). We differentiate here the number of ARG per genome vs the number of ARG per genome multiplied by the relative abundance of the species. It is very important to take both aspects into consideration, as some species can carry a lot of ARGs while being very low in relative abundance, while some species have few ARGs but are highly abundant. To consider these 2 aspects, we looked at species using both cpm normalization and raw ARG count (i.e., raw number of ARGs per MAG). In both cases (Figure 4b,c), we found that *Escherichia coli* is the top ARG carrier/abundant bacteria overall for most countries. Species belonging to the genera *Bacillus, Citrobacter, Enterobacter, Erwinia, Escherichia, Klebsiella, Pseudomonas*, and *Staphylococcus* were the top ARG carriers/abundant bacteria for most countries. Most of these genera carrying high ARG abundance are clinically relevant pathogens and are involved in a wide variety of infections including but not limited to nosocomial infections and intestinal infections (https://www.hartmann-science-center.com/en/hygiene-knowledge/pathogens-a-z).^34^

Some species such as *Escherichia albertii*, *Escherichia ruysiae*, and *Klebsiella michiganensis* only appear when looking at the number of ARGs per genome without taking the relative abundance into account. Interestingly, the top species differ from Figures 4b–c for each country, as reflected by a slightly different country clustering between the 2 figures. For example, for New Zealand, the top species is *Citrobacter youngae* in Figure 4b while it is *Escherichia marmotae* in Figure 4c.

Furthermore, inspecting the top 5 species per country carrying the highest ARG abundance (Figure 4b), we report that species belonging to the genera *Escherichia, Enterobacter and Klebsiella* were in this category for most countries, while species belonging to the genera *Pseudoescherichia* and *Bifidobacterium* were present in some countries such as New Zealand, the US, and the UK. An interesting result was the presence of *Bifidobacterium* in the top 5 cpm normalized but not in raw abundance data (i.e., number of genes per genome) (Figure 4b,c). This is attributed to the high abundance of this genus in early life and the presence of several intrinsic resistance genes.^35^

**Figure 5:**
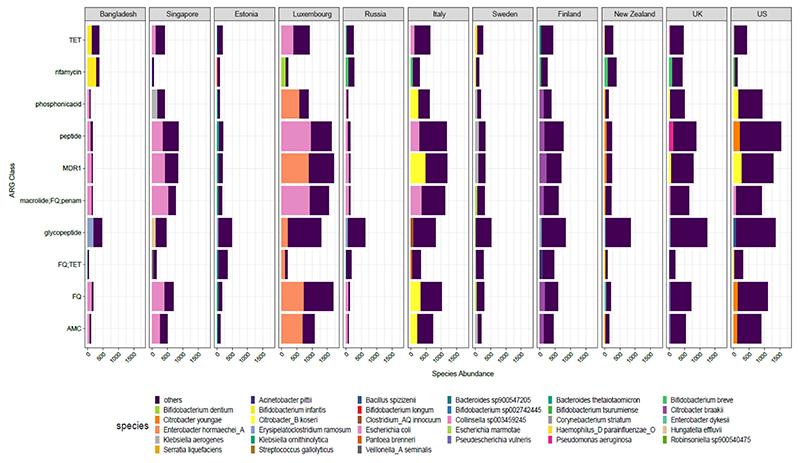
Bar plot displays the average top 5 ARG in cpm for each country along with the proportion contribution of the top 1 bacteria. Rest of the species are denoted as “others”. The abbreviations used for ARG classes are as follows: MDR1 = “FQ; cephalosporin; glycylcycline; penam; TET; rifamycin; phenicol; disinfectingagentsandantiseptics”; TET = “Tetracycline”; AMC = “aminocoumarin” and FQ = “fluoroquinolone”.

We then examined the top 5 most abundant antibiotic classes per country and the proportion of top bacterial species carrying these classes (Figure 5). The most abundant antibiotic resistance classes and species results were consistent with the above results, with the top species belonging to the genera *Bifidobacterium, Citrobacter, Erysipelatoclostridium, Escherichia, and Pseudomonas* seen in several countries examined. As observed above (Figure 4b) the highly abundant ARG carrier was *Enterobacter hormaechei* in Luxembourg, *Citrobacter youngae* and *koseri* spp. in the US and *Escherichia coli* was high in several countries including Bangladesh, Singapore, and Luxembourg. The antibiotic classes included glycopeptide, fluoroquinolone, tetracycline, and rifamycin, among others, along with MDR classes.

### Antibiotic resistance class relative abundance and diversity varies by age of life, country, continent, socioeconomic status and HAQ index

**Figure 6:**
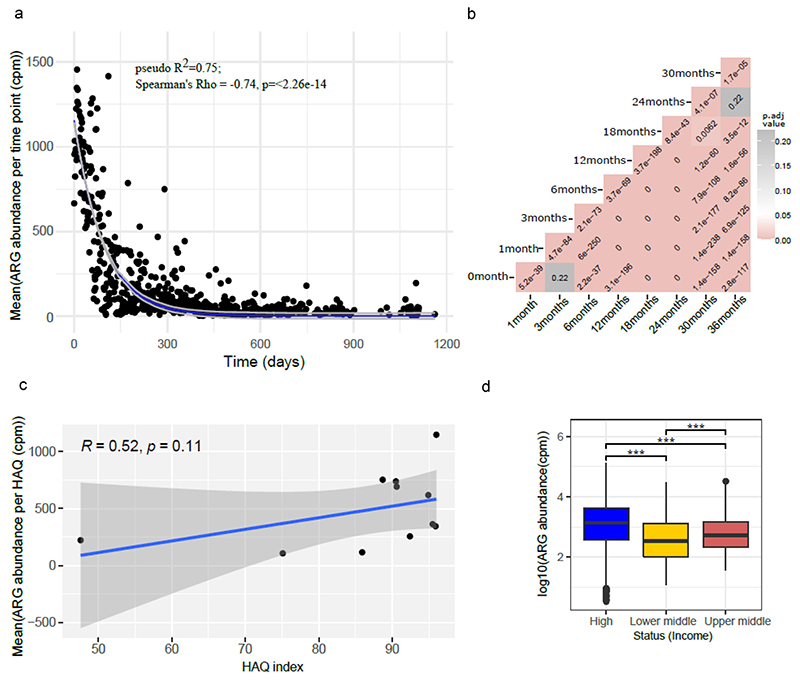
(a) Plot showing exponential decay curve demonstrating the relation between ARG abundance in cpm and age shown as time in days (pseudo R-squared = 0.75, AIC = 8392.51). Gray area denote confidence intervals. (b) Heatmap showing adjusted *p-*values (adjusted using Bonferroni Hochberg method) obtained from Wilcoxon test comparing time points pairwise, with the formula arg_abundance ~ month with ggpubr package in R. (c) Plot showing association between log transformed ARG abundance normalized to cpm and Healthcare access and quality (HAQ) index (d). Box plot showing relation between log transformed ARG abundance in cpm and socio-economic status of countries. *p*-value ≤ 0.05; ** *p*-value ≤ 0.01; *** *p*-value ≤ 0.001. Significance was determined using the ggpubr package in R using Wilcoxon test.

We assessed the relationship between ARG class abundance and infant age in months, socioeconomic status, and the HAQ index (Figure 6). Significant differences in the abundance of antibiotic resistance classes were found based on these variables. In general, a higher abundance of ARGs was observed in very early life, which decreased with time showing an exponential decay (pseudo-R^2^ = 0.75, AIC = 8392.51) (Figure 6a,b). While the lowest ARG abundance was observed in countries corresponding to a very low HAQ index (Figure 6c), we did not observe any significant correlation between ARG abundance and the HAQ index using Spearman’s correlation (*p*-value = 0.11). Nonetheless, ARG abundance was significantly different between country’s socioeconomic status, with low- and middle-income countries having low HAQ (Figure 6d) (Wilcoxon test, adjusted *p*-value < 0.001).

**Figure 7:**
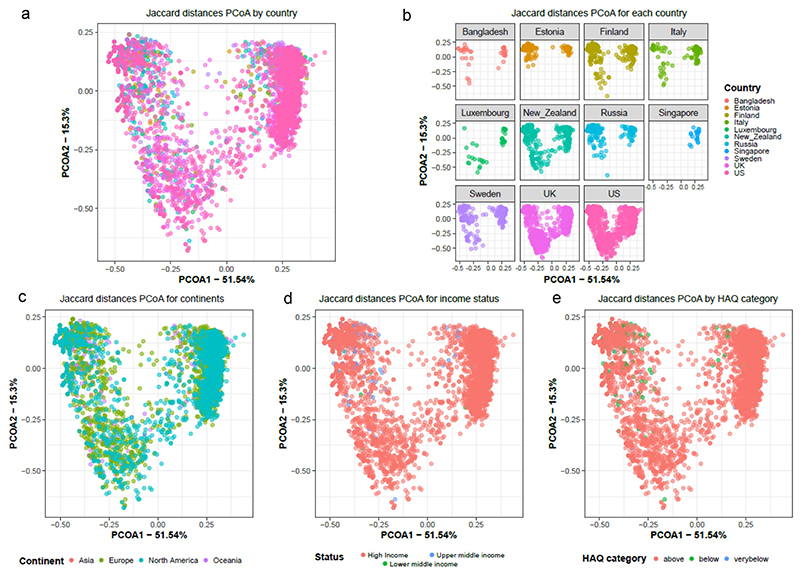
Beta diversity as represented using PCoA computed using Jaccard distances showing grouping by (a) country, (b) for each country, (c) continent, (d) socio-economic status and (e) HAQ index category. All plots show the same PCoA computed with the same distance matrix.

To assess the relationship between the composition of antibiotic resistance classes and the study variables, beta diversity analyses were performed using Jaccard distances, taking time into account both as a potential cofounder and repetitive measurement (Figure 7). The results show that the ARG class composition is significantly different between countries (*p*-value = 0.001, PERMA NOVA), socioeconomic status of the country (*p*-value = 0.001, PERMANOVA), continent (*p*-value = 0.001, PERMANOVA), and HAQ index (*p*-value = 0.001, PERMANOVA). Pairwise PERMANOVA further indicated that ARG composition between each 2 continent comparisons was significantly different (adj. *p*-value < 0.05) apart from Asia vs Europe (Table 1). Further, we looked at the ARG composition with respect to time in months and observed significant differences between months and also between countries for each month (Figure S2A and S2B; *p*-value = 0.001; PERMANOVA).

### Antibiotic efflux is the most common resistance mechanism in infants

**Figure 8:**
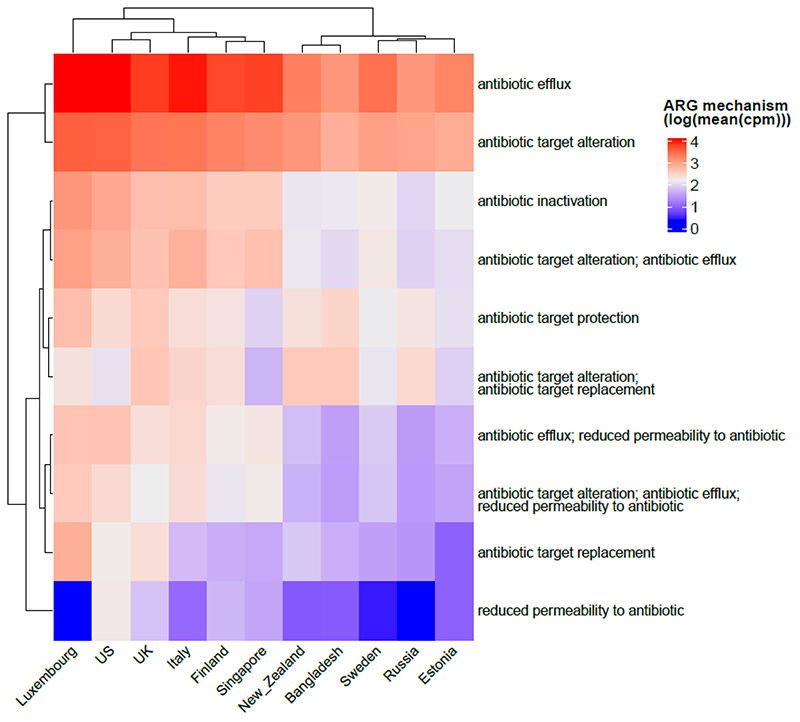
Heatmap showing common antibiotic resistance mechanisms observed in all the countries included in our meta-analysis. The abundance is shown as log of mean of normalized cpm values.

The antibiotic resistance mechanisms that are most prevalent in infants under 3 years of age across populations in our study were investigated. Antibiotic efflux (intrinsic) was the most common mechanism of resistance, followed by antibiotic target alteration and inactivation (both can be acquired), overall and for each country (Figure 8). Reduced permeability to antibiotics, on the other hand, is the least present ARG mechanism, and is surprisingly not present in Luxembourg despite this country presenting one of the highest diversity of antibiotic resistance classes.

## Discussion

The rise and spread of AMR is many times higher than antibiotic consumption and can be attributed to over- and misuse of antibiotics and globalization. Recent studies have monitored the global ARG profile based on various sampling sites including sewage, toilet waste and various habitats.^36–38^ However, to the best of our knowledge ours is the first meta-analysis to examine the resistome profile in early life on an international scale. In this meta-analysis, resistance to several drug classes categorized as “reserve” and “watch” by the WHO was observed to be high in all the countries. These antibiotic classes are suggested to be used as last-resort antibiotics; thus, the presence of resistance to reserve category antibiotics in early life is alarming. We also report an exponential decay of ARG abundance (cpm) in infant gut as the infant ages. This is complementary to what was reported by Zeng et al.,^1^ showing a decrease of ARG richness per MAG with time. We also observed that species carrying the most abundant ARGs are clinically relevant in infants and belong to the genera *Escherichia, Klebsiella, Enterobacter*, and *Pseudomonas*.

From the countries included in our study, as per the 2016 World Bank classification, only Bangladesh was classified as a Lower-middle-income and Russia as an upper-middle-income country, while all other countries were high-income (https://datatopics.worldbank.org/world-development-indicators/the-world-by-income-and-region.html). These socioeconomic statuses translate to the HAQ status, with Bangladesh having the lowest HAQ (47.6), and Russia being the second with 75.1 in 2016 among the countries included in our study.^39^ We observed a difference in abundance and diversity of antibiotic resistance classes based on these variables with a low HAQ index showing lower relative ARG abundance, without any significant correlation between HAQ index and ARG abundance. In our study, a similar relationship was observed in terms of socioeconomic status, where high-income countries including Luxembourg, the US, and the UK showed higher relative ARG class abundance and diversity, followed by middle-income countries. This may be because people in high-income countries have easier access to antibiotics. Our results with regard to ARG abundance and socioeconomic status are not in line with Hendriksen et al.^37^ and this could be due to the different approaches used in the study design (sewage samples vs infant gut metagenomes) and also the difference in the geographical coverage and sampling times in the 2 studies. Furthermore, previous studies have reported a difference in antibiotic consumption profiles in low- and high-income countries, with low- and middle-income countries showing a high and increasing trend in the early 2000s. The rate of antibiotic use was seen to increase from to 2010–2015 in some low-middle-income countries, but it varied by antibiotic type, and the overall global rates were high.^40–42^ A high rate of antibiotic consumption has also been reported in high-income and upper-middle-income countries.^43^ The socio-demographic status of a country has been shown to be associated with HAQ^39^ and a small increase in the misuse of antibiotics is observed in middle- and high-income countries.^44^ These differences in rates of antibiotic consumption in various countries over time also explain the differing results of our study from other large-scale resistome studies by Hendriksen et al.^37^ and Munk et al.^45^

Furthermore, an exponential decrease in ARG class abundance with increasing age was identified; this can be linked to the development of dynamic infant gut microbiota in early life. The species with the highest ARG abundance in our study were gram-negative and belonged to the genera *Citrobacter, Escherichia, Enterobacter, Pseudomonas*, and *Klebsiella* which are all known pathobionts and are responsible for causing sepsis in infants.^46^ These genera were consistently detected to carry relatively high ARG abundance in our analysis when looking at the number of ARGs per genome, and a combination of number of ARGs per genome and relative abundance of the corresponding species. This points to the fact that species carrying high number of ARGs are also usually abundant, making these pathobionts multi-drug-resistant bacteria. Nonetheless, some bacteria differ depending on these 2 approaches. For example, *Bifidobacterium* species were remarkably high in ranking when looking at the combination of ARG abundance and number of ARGs per genome, which is surprising and should be further studied. Reciprocally, some species of bacteria, such as *Escherichia albertii* or *Klebsiella grimontii*, carry a high number of ARGs but are not very abundant, making them pathobionts potentially difficult to detect. High number of ARGs could also be related to a higher probability to share these genes with other gut microbiome bacteria through horizontal gene transfer, especially with closely related species of the same genus.

A certain advantage of the current meta-analysis is that it uses the most updated set of MAGs generated from infants in early life – the global early life gut genome (ELGG) reservoir generated as a part of previous meta-analysis consisting of data from multiple countries.^1^ Our analysis adds to our previous study^1^ but is independent of antibiotic use or health status of the individual, which would enable a comprehensive overview, as these meta-data were absent in many cases. Though our analysis might not be directly useful for the creation of antimicrobial stewardship programs, it will help form the basis of further studies to look into localized resistome profiles in the country to gain deeper insights. Certain limitations are that, as MAGs are generated only from shotgun sequencing data, we were restricted to the number of studies/countries that passed the inclusion criteria to be included in the analysis. This resulted in many data gaps due to the lack of datasets from many low- and middle-income countries. Another limitation of the study is the fact that socioeconomic variables can vary in large countries from one geographical location to another.^39^ However, we could not consider this factor in our analysis as our meta-analysis was focused on examining resistome profiles from ELGGs generated as a part of our previous study (Zheng et al., 2022) which included 26 studies based on the search criteria. Furthermore, the depth of sequencing, longitudinal sample collection, and varying time durations of the studies are all factors that restrict the analysis.

In conclusion, antibiotic resistance was high in infants in all countries included in the meta-analysis and was associated with the geography, age, and demographics of the country. Further studies that combine antibiotic use and resistance data in infants are needed to limit the use of antibiotics in early life. Based on our analysis, we understand that the resistome profile in early life is not similar across populations and differences exist in countries; with further analysis needed to understand the differences within countries. The presence of specific antibiotic stewardship programs reduces the use of antibiotics in NICUs.^9^ This, along with the high resistance rates reported and the high use of antibiotics due to the COVID-19 pandemic,^47^ emphasizes the need for stricter guidelines and updated antibiotic stewardship programs that can be location specific and be based on a regularly updated database of ARG presence in individuals. Irrespective of whether the final aim is location-specific antibiotic stewardship programs or studying the resistome profile of individuals with respect to antibiotic use, a special focus must be made on infant groups in various geographic locations due to the high importance of microbiota in early life forming the foundation for later life.

## Methods

### Publicly available data selection and retrieval

**Figure 9:**
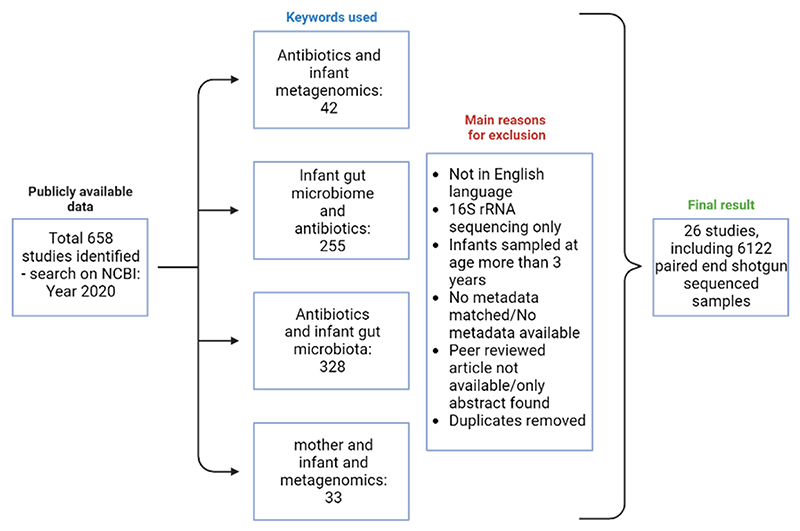
Diagrammatic representation of the study design delineating the inclusion and exclusion criteria for studies included in the meta-analysis. Figure was created using Biorender.

The PubMed database was searched using NCBI to identify studies including shotgun sequencing data generation/analysis in 2020. This search was restricted to June 2020 and was carried out using the following keyword combinations: “antibiotics and infant metagenomics”, “infant gut microbiome and antibiotics”, “antibiotics and infant gut micro-biota, and “mother and infant metagenomics”. The inclusion of papers was limited to papers published in English and peer-reviewed, and those that provided metadata about the metagenome samples (Figure 9). This filtering along with duplicate removal resulted in 26 papers matching the inclusion criteria, resulting in 6122 metagenome samples that were matched to the metadata from each paper. The metadata extracted from each paper included delivery mode, infant, and maternal antibiotic information, day of life of sample collection, sex, accession ID, and country of study. The shotgun sequencing data for the 6122 samples were downloaded using NCBI SRA by matching the accession IDs published for each study using Prefetch. Fastq-dump with “–split-3” from SRA tools (v2.9.2_1) was used to convert the downloaded SRA files to paired-end FASTQ files.

### Data processing and antibiotic resistance analysis

FASTQ files were first trimmed and filtered to remove any human host contamination (Homo sapiens database) using KneadData (v0.7.2) with default parameters. Quality-controlled reads were then concatenated and used for taxonomic classification using Metaphlan3 as part of the Humann3 run.^48^

The 6122 metagenomes filtered reads were used for generation of metagenome-assembled geomes (MAGs), resulting in the the formation of 32,277 early life gut genomes (ELGG).^1^ The process used for generation of MAGs is described in detail in Zeng et al.^1^ Briefly, the filtered reads from 6122 metagenomes were assembled using MegaHIT (v1.1.3) with option “-min-contig-len 1000”, which resulted in 29,912,553 contigs. The MAGs were generated per sequencing run using three metagenomic binning tools (MetaBAT v2.12.1, MaxBin v2.2.624, and CONCOCT v1.0.025) using metaWRAP (v1.3.1) with default parameters. The minimal contig size for binning was set as default with 1000 bp (except for MetaBAT2, which required at least 1500 bp). The resulting bins were refined using Bin_refinement module of metaWRAP with options “-c 50 -x 10”. CheckM (v1.0.12) was used to determine the quality of bins. Taxonomic annotation of the resulting MAGs was performed using GTDB-Tk (v2.1.0). The resulting 32,277 genome assemblies are available online and were used for resistome analysis. To examine the ARG profile globally in the infant gut, RGI main (v6.0.0) with CARD database^49^ was used, using default parameters on the MAGs and considered only “perfect” and “strict” hits for further analysis. The quant_bin module of the metaWRAP (v1.3.1) tool was used to obtain genome copies per million reads (relative abundance) for each MAG per sample. To obtain gene copies per million reads (cpm) for each antibiotic resistance gene for each MAG, we multiplied the gene count per MAG by the MAG cpm values. Throughout the study, we used average cpm values per sample for each gene (for each sample or for each variable or group of variables considered), calculated from the gene cpm values for each MAG.

### Bioinformatics and statistical analysis

Results from RGI were either used directly as count/richness of ARGs/antibiotic classes per MAG, or after normalizing to copies per million (cpm) using MAG relative abundance (in cpm). HAQ index data for 2016 were obtained from a previous study^39^ and socioeconomic status data for the countries for the year 2016 were obtained from the World Bank (https://datatopics.worldbank.org/world-development-indicators/the-world-by-income-and-region.html) (Table 2). The HAQ index provides information about gains and gaps in personal health-care access and quality in a country. The HAQ index was obtained on a scale of 1–100 and for further analysis it was transformed into the following categories: very low/below (≤50), low/below (≤80), and above (>80). Downstream processing of the results along with visualization was performed in R using several packages, including ggtree, maps, ggplot2, complexHeatmap, vegan, and scatterpie. Statistical analysis was performed in R using the pairwiseAdonis, ggpubR, and ggsignify packages. PERMANOVA and pairwise PERM ANOVA were performed using the ‘adonis2’ and ‘pairwiseadonis’ functions in R. To account for repetitive measurements, we used the ‘strata’ option in adonis2. To account for time as a potential cofounder, we grouped the samples by month categories (0, 1, 3, 6, 12, 18, 24, 30, 36 months) and added it in adonis2 for all comparisons (e.g., ~ country + month). To perform pairwise comparison, as mentioned in the text, we used the Wilcoxon test. The relationship between age (day of life) and averaged ARG abundance per time was modeled using an exponential decay function:

(1) $$ARG(t)=a\cdot e^{-bt}$$

where ARG(t) is the number of ARG at a given time t, a and b are positive parameters. To fit the parameters of (1), we used the ‘nls’ function of R. Asymptotic confidence for expected response intervals were calculated as described previously (https://www.jchau.org/2021/07/12/asymptotic-confidence-intervals-for-nls-regression-in-r/). To assess the goodness of the fit, we used pseudo R-squared and AIC criteria. Spearman’s correlation coefficient was used to determine the correlation between ARG abundance and study variables such as age, socioeconomic status, and HAQ index. Throughout the study, statistical significance was determined by 999 permutations for PERMANOVA, and p-values were corrected using the Benjamini–Hochberg method. Additionally, p.adjusted values below 0.05 were considered significant.

## Supplementary Material

Supplementary figures

## Figures and Tables

**Table 1:** Results from PairwiseAdonis test in R showing *p-*value and p.adjusted values for comparison for beta diversity analysis.

HAQ category					
pairs	Df	R2	p.value	p.adj	sig
verybelow vs below	1	0.00184	0.002	0.006	*
verybelow vs above	1	0.001284	0.014	0.042	.
below vs above	1	0.007512	0.001	0.003	*
**Status**					
Lower middle income vs high income	1	0.000884	0.002	0.006	*
Lower middle income vs upper middle income	1	0.03647	0.001	0.003	*
High income vs upper middle income	1	0.024864	0.001	0.003	*
**Continent**					
Asia vs Europe	1	0.001535	0.01	0.06	
Asia vs Oceania	1	0.020091	0.001	0.006	*
Asia vs North America	1	0.002026	0.002	0.012	.
Europe vs Oceanic	1	0.026171	0.001	0.006	*
Europe vs North America	1	0.013297	0.001	0.006	*
Oceania vs North America	1	0.056484	0.001	0.006	*

p adjusted values below 0.05 are considered significant and marked with * or. in the sig column.

**Table 2:** Table displays the socio-economic status and Health care access and quality index score for all the countries included in our analysis.

Country	Low-middle or High income (World bank classification 2016)	Healthcare access and quality (HAQ) index – for 2016, absolute change from 2000–2016
Bangladesh	Lower middle	47.6 (44.3–50.9), 20.1 (16.3–23.8)
Estonia	High	85.9 (83.6–88.3), 14.3 (11.8–17.0)*
Finland	High	95.9 (94·6–96.9), 8.1 (6.7–9.5)
Italy	High	94.9 (93.4–96.0), 6.1 (4.7–7.4)*
Luxembourg	High	96.0 (94.4–97.3), 5.7 (3.9–7.4)*
New Zealand	High	92.4 (90.3–94.3), 5.4 (3.1–7.4)
Russia	Upper middle income	75.1 (67.7–81.7), 12.6 (5.0 to 19.4)*
Singapore	High	90.6 (87.2 –93.3), 10.9 (7.1–14.8)*
Sweden	High	95.5 (93.4–97.2), 3.1 (1.0–5.0)*
UK	High	90.5 (89.6–91.3), 6.5 (5.9–7.2)*
USA	High	88.7 (88.0–89.4), 1.9 (1.4–2.5)*

## Data Availability

No new data were generated as part of this study, and previously published publicly available data were used for the analysis.
