# Supplementary material for: Influence of age, socioeconomic status, and location on the infant gut resistome across populations
Source: Gut Microbes. Author manuscript; Available in PMC 2024 Jan 29. (PMC10793692; doi:10.1080/19490976.2023.2297837)
Supplement: Supplementary figures [file EMS193426-supplement-Supplementary_figures.docx]

**Figure S1:**


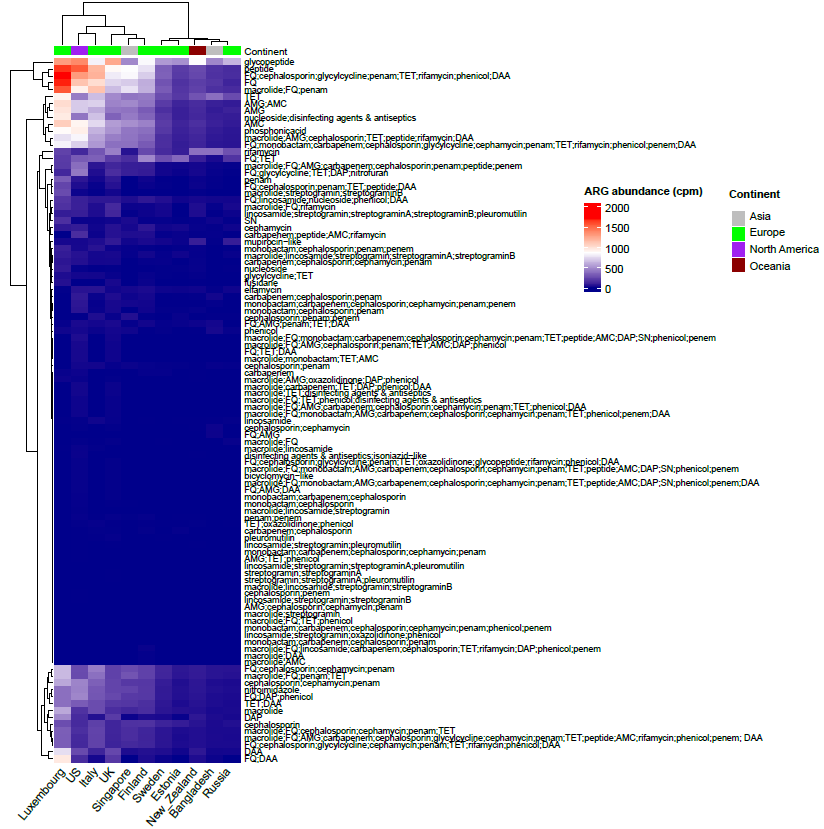


Figure S1: Heatmap showing all the classes of antibiotics per country. Columns in the heatmap represent countries while rows represent antibiotic classes with top row annotation showing continents. Some of the antibiotic classes are abbreviated in the heatmap and are as follows: AMG = “aminoglycoside”, TET = “tetracycline”, RIF = “rifampin”, FQ = “fluoroquinolone”, DAA = “disinfecting agents and antiseptics”, DAP = “diaminopyrimidine”, SN = “sulfonamide” and AMC = “aminocoumarin”.

**Figure S2:**


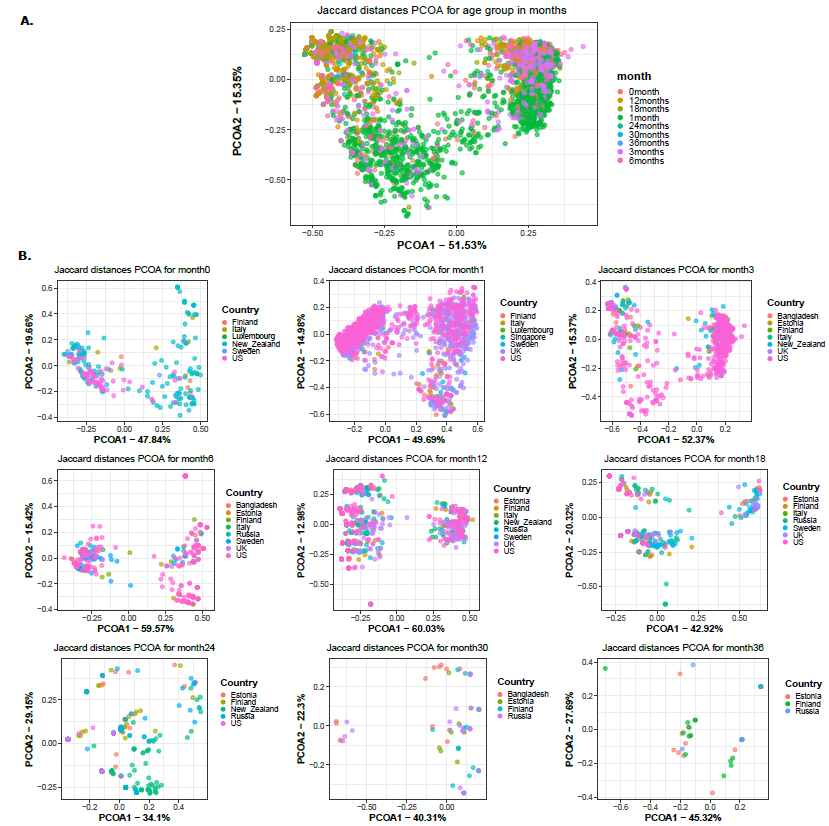


Figure S2: Beta diversity as represented using PCoA computed using Jaccard distances showing grouping by A. all months and B. individually subsetted month where the data points are coloured by the country.
